# Development of an Artificial Neural Network Utilizing Particle Swarm Optimization for Modeling the Spray Drying of Coconut Milk

**DOI:** 10.3390/foods10112708

**Published:** 2021-11-05

**Authors:** Jesse Lee Kar Ming, Mohd Shamsul Anuar, Muhammad Syahmeer How, Samsul Bahari Mohd Noor, Zalizawati Abdullah, Farah Saleena Taip

**Affiliations:** 1 Department of Process and Food Engineering, Faculty of Engineering, Universiti Putra Malaysia, Serdang 43400, Malaysia; jeslkm@yahoo.com (J.L.K.M.); mshamsul@upm.edu.my (M.S.A.); syahmeerhow@upm.edu.my (M.S.H.); 2 Department of Electrical and Electronic Engineering, Faculty of Engineering, Universiti Putra Malaysia, Serdang 43400, Malaysia; samsul@upm.edu.my; 3 School of Chemical Engineering, College of Engineering, Universiti Teknologi MARA, Shah Alam 40450, Malaysia; zalizawati8653@uitm.edu.my

**Keywords:** spray drying, coconut milk, artificial neural network, particle swarm optimization, processes

## Abstract

Spray drying techniques are one of the methods to preserve and extend the shelf-life of coconut milk. The objective of this research was to create a particle swarm optimization–enhanced artificial neural network (PSO–ANN) that could predict the coconut milk spray drying process. The parameters for PSO tuning were selected as the number of particles and acceleration constant, respectively, for both global and personal best using a 2^k^ factorial design. The optimal PSO settings were recorded as global best, C_1_ = 4.0; personal best, C_2_ = 0; and number of particles = 100. When comparing different types of spray drying models, PSO–ANN had an MSE value of 0.077, GA–ANN had an MSE of 0.033, while ANN had an MSE of 0.082. Sensitivity analysis was conducted on all three models to evaluate the significance level of each parameter on the model, and it was discovered that inlet temperature had the most significant influence on the model performance. In conclusion, the PSO–ANN was found to be more effective than ANN but less effective than GA–ANN in predicting the quality of coconut milk powder.

## 1. Introduction

The unit operation of spray drying is defined as a process that converts a liquid feed into a powder form. In comparison to similar drying processes, spray drying processes have better industrial advantages in terms of larger output design and compatibility with a variety of heat-sensitive and -resistant materials. It is a complicated process that requires the use of fundamental engineering knowledge to relate the processing parameters to product characteristics [1]. Furthermore, feed characteristics, such as viscosity, glass transition temperature, and agglomerations, make process modeling, control, and optimization more challenging. Mechanistic models can provide useful insights into the spray drying process, but developing them is a huge challenge [2].

Owing to the short lifespan of coconut milk, many processing and preservation methods have been investigated. They have shown potential viability, but have limits in terms of processing costs, shelf-life duration, and consumer safety [3,4,5]. Spray-dried coconut milk power offers a better packing value and shelf-life than other coconut milk derivatives [6]. Coconut milk has a low melting point, ascribed to a larger percentage of short chain triglyceride content. As a result of the high spray drying temperature and the low glass transition temperature of coconut milk, numerous setbacks in spray drying production have occurred, including adhesion of coconut milk powder to the wall, which often leads to lower yield and poor storage and handling [7].

The development of models for the spray drying process is challenging because of the complexity of the process. The interdependence between the variables (independent and dependent) in the system also influences the complexity of the spray drying process, resulting in different powder quality, especially in moisture content, particle size, flowability, and others. In many studies, it was found that inlet temperature and feed concentration had a statistically significant effect on powder moisture content [8].

Data-driven models, such as ANN, are an effective modeling technique for spray drying processes. The ANN corresponds to and simulates the learning process using experimental data provided and it determines the connection between the variables involved [9]. Furthermore, the process of ANN development requires fewer fundamental theories on spray drying mechanisms to link the relationship between input variables (operating parameters) and output variables (powder properties) [10,11]. ANN has been used in a variety of food processing applications, such as paddy drying, lactose drying, cocoa bean processing, and others [12,13,14]. ANN has been applied and showed good performance in the spray drying of orange juice [15], pomegranate [16], rhubarb juice powder [17], and olive oil [18].

The neural weight selection is a critical component of the neural network development, especially in the training phase. In this phase, the neural weight is proposed to be optimized as each weight of the neural connection to be established and build a network with parameters that can produce accurate output [19]. The advantages of this method are to ensure solutions in the neural network search space can escape local minima points and better convergence in locating global minima points [20]. Studies on the drying of bayberry fruit and fluidized bed drying of onions emphasized that prior selection of the neural network training algorithm is important to improve the model’s accuracy [21,22].

PSO is a population-based stochastic optimization algorithm that is based on the movement of animal behaviors attributed by Eberhart Kennedy [23]. The PSO algorithm mimics this movement by generating a swarm of particles that move around in a search space, to achieve a set of goals that are dictated by a fitness function (cost), to achieve an optimal solution. This function measures particle fitness by adjusting each particle location depending on its own personal best experience and the best particle position of the swarm [24]. PSO has been used in food-related optimization; for example, in modelling the mastication of white rice [25]. The integration of PSO and ANN has been utilized in the drying of guava pieces [26] as the use of optimization techniques led to a significant improvement in the ANN. The study showed that the optimized neural network employing PSO obtained almost 99% recognition accuracy in terms of R^2^ values, whereas the ANN models achieved 86%. The innovation of this study involved optimizing PSO parameters and ensuring that hybrid ANN can performed in a robust structure under a well-designed experiment. The PSO was applied to the feedforward neural network as a global search algorithm, where the position of each particle represents a set of weights for the current iteration. The algorithm’s goal is to minimize error as the particle moves within the search space from its original position.

The aim of this study was to create an ANN of the coconut milk spray drying process integrating the PSO technique. RSM was used to improve the chosen parameters of the PSO optimization technique, which was then incorporated into the creation of the ANN through weight initialization.

## 2. Materials and Methods

### 2.1. Framework Study

**Figure 1:**
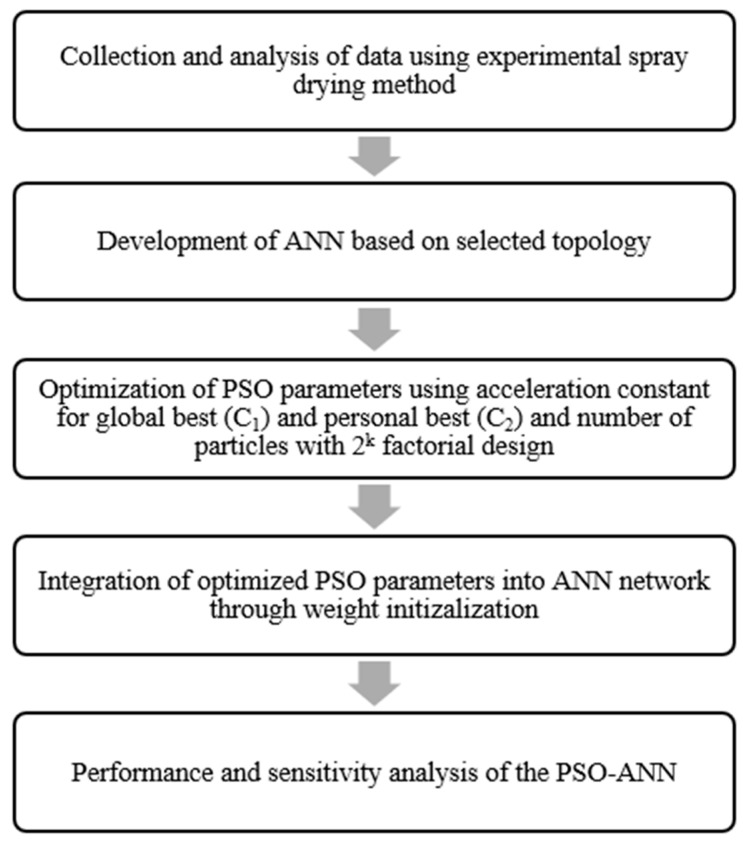
Research framework for PSO development.

According to Figure 1, the PSO–ANN development process is made up of consecutive methodological steps. First, experimental spray drying data of selected parameters were collected, and the quality of coconut milk powder was examined. The neural network was constructed using various topology designs based on experimental data and consisted of three inputs (inlet temperature, concentration of maltodextrin, and concentration of sodium caseinate) and three outputs (outlet temperature, moisture content, and surface free fat). PSO parameters were optimized using a 2^k^ factorial design, with the parameters selected for PSO tuning being the number of particles and the acceleration constant for both global and personal best. PSO optimization techniques were integrated into the ANN to determine the optimum weights in the neural network design using MATLAB version 2019a. Lastly, the developed PSO–ANN was compared with external ANN [27] and GA–ANN [28] based on MSE and R^2^ evaluation and supported with sensitivity analysis.

### 2.2. Coconut Milk Powder Production

Coconut milk emulsion was prepared using fresh coconut milk homogenized with two different types of additives, maltodextrin and sodium casein, followed by spray drying using a laboratory spray dryer (SD-05). The spray drying process was carried out using different values of temperature (140, 150, 160, and 170 °C), concentration of maltodextrin and sodium caseinate (between 0 *w*/*w*%–10 *w*/*w*%). The coconut milk powder was kept and sealed for analysis.

### 2.3. Moisture Content

The moisture content of coconut milk was measured by placing two grams of spray dried coconut milk powder in a moisture analyzer MX-50 (A&D Weighing, San Jose, CA, USA) (Equation (1)). The measurement of moisture percentage was performed in triplicate, and the average was calculated.

(1) $$Moisture=\frac{b-c}{b-a}\times 100\%$$

where *a* = the weight of empty dish, *b* = the weight of dish + powder, and *c* = the weight of dish + dried powder.

### 2.4. Surface Free Fat

Twenty-five milliliters of petroleum ether was mixed with 2 g of spray-dried powder and shaken for 10 min using a vortex mixer. The solution was then filtered using a filter paper as the powder separated owing to density difference and the powder collected on the filter paper. The powder was further evaporated in a hot water bath to remove the remaining petroleum ether. The residual powder was then dried in an oven at 102 °C until constant weight was achieved. The weight was measured in triplicate and the average was determined.

### 2.5. Outlet Temperature

The outlet temperature was measured in triplicate and the average outlet temperature (°C) was calculated. The outlet temperature was the temperature taken at the outlet powder source of the spray dryer system where the performance of the outlet temperature reading was dependent on the inlet temperature, air humidity, and the air flow rate.

### 2.6. Development of Artificial Neural Network

The ANN used is a multilayer perceptron (MLP) neural network with optimum configuration with a topology of 3-2-8-3, transfer function of logsig, and a Levenberg–Marquardt algorithm using K-Fold cross validation, which was established from research conducted by the previous authors [27]. Using normalized data, the optimal ANN design is determined using a selected network topology design, such as number of neurons (5–15), hidden neural layers (1–3), four different transfer functions, and seven training algorithms. The most efficient topology was obtained through trial-and-error selection of various network designs using two criteria that were based on mean square error (MSE) and R^2^ value.

### 2.7. PSO Algorithm Development

**Figure 2:**
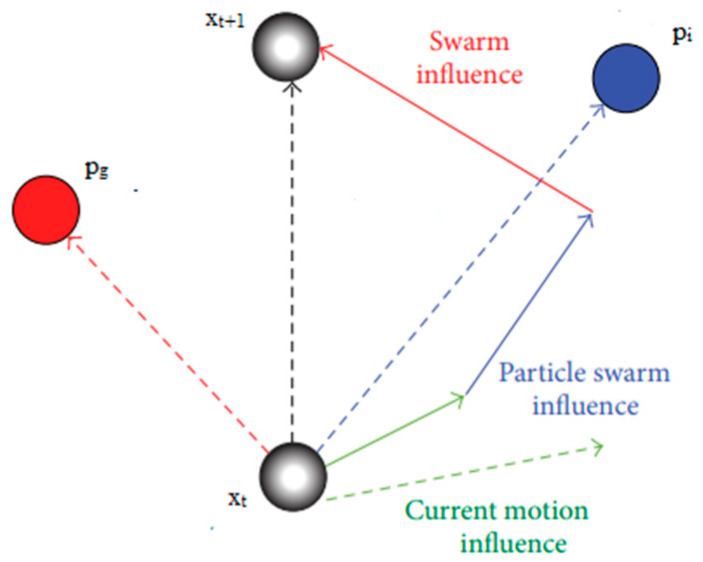
Illustration of particle’s position after being subjected to PSO.

The PSO algorithm initializes a set of random particles to determine the best solution in the search space through a designated iterative means. Each particle has its own velocity, which repeatedly updates based on two main factors: its individual best position from its main original position (*p_i_*) and the individual best position (*p_g_*) from the overall global best position of the entire swarm. Therefore, at each (*t* + 1)th iteration, the particle’s position and velocity are calculated using the following equations:

(2) $$\upsilon_{i}\left(t+1\right)=\omega \upsilon_{i}\left(t\right)+c_{1}r_{1}\left(p_{i}\left(t\right)\right)-x_{i}\left(t\right))+c_{2}r_{2}\left(p_{g}\left(t\right)\right)-x_{i}\left(t\right))$$

(3) $$x_{i}\left(t+1\right)=x_{i}\left(t\right)+v_{i}\left(t+1\right)$$

where $\upsilon_{i}$ and $x_{i}$ are the velocity vector and the position vector of particle *i* at t-iteration, respectively. The cognitive and social parameters are represented by specific acceleration parameters, *c*_1_ and *c*_2_. Both parameters are focused on retaining particle control movement in search space, as they both control the influencing balance of the personal best and global best particle positions. *r*_1_ and *r*_2_ are random numbers in the interval [0,1] generated by the uniform distribution function. ω is the inertia weight parameter that is used as a velocity constraint mechanism. The value represents the size of space exploration or search region. Figure 2 illustrates the position of *x_t_*_+1_ from *x_t_* after it has been subjected to the PSO.

### 2.8. Optimization of PSO Parameters

Design of experiments (DOE) was performed using MINITAB version 17 to further optimize the PSO settings using a 2^k^ factorial design, where the numerical value of two represented the two levels of k factors. Each parameter’s values were set to be low and high. The DOE implementation was based on the PSO optimization parameter, in which significant inferences and reasoning was attained from analyzing the opposing values [29]. Table 1 summarizes the parameters that were optimized and their significance [23,30,31,32].

### 2.9. PSO Integration into ANN Development

**Figure 3:**
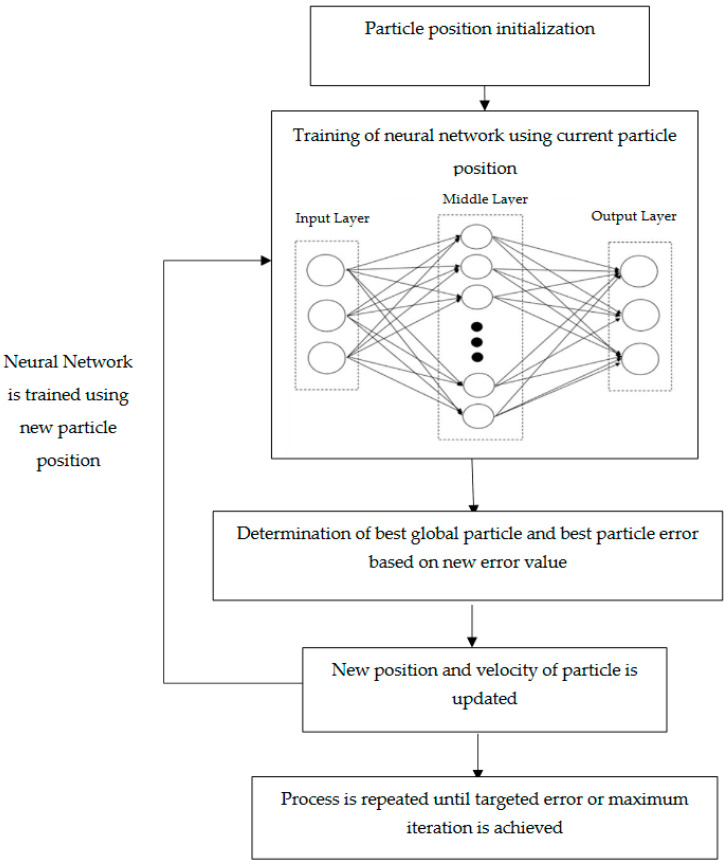
PSO learning algorithm in ANN development.

The PSO is applied to the feedforward neural network as a global search algorithm, where the position of each particle represents a set of weights for the current iteration. The algorithm’s goal is to minimize error as the particle moves within the search space from its original position. As illustrated in Figure 3, the particle’s dimensionality resembles to the number of weights given to the network. As the particle moves from one position to another at a certain velocity and epoch, the weight associated with the particle changes to achieve the objective of the algorithm. The new position represents a new set of weights that represent a new error. At each epoch, the particles continuously update their position by taking their velocity and various factors into account in the algorithm. The process is repeated until the PSO algorithm achieves the desired error value, or the maximum iteration is reached.

### 2.10. Cost Function

The PSO optimized the cost function through a population-based search, in which the assigned PSO parameters project each particle towards a region that has the lowest MSE value function. The goal of optimizing neural network weights and MSE was based on a cost function [20], which is described generally as:

(4) $$C\left(w,b\right)=\frac{1}{2n}\underset{x}{\sum }\|y\left(x\right)-{a\|}^{2}$$

where *w* represents the weights in the network, *b* is biases, *n* is the total number of training inputs, *a* is the vector of outputs from the network when *x* is the input, and *y*(*x*) is the sum of inputs of *x*.

### 2.11. Performance Comparison of ANN and PSO–ANN

The performance of both ANN and PSO–ANN was compared using correlation of determination, R^2^, and MSE. The formulas for the criteria are as follows:

(5) $$\mathrm{MSE}=\frac{1}{N}\overset{n}{\underset{i=1}{\sum }}{\left(T_{p,i}-T_{a,i}\right)}^{2}$$

(6) $$R^{2}=\sqrt{\frac{\sum_{i=1}^{N}{\left(T_{p,i}-T_{a,average}\right)}^{2}-\sum_{i=1}^{N}{\left(T_{a,i}-T_{p,i}\right)}^{2}}{\sum_{i=1}^{N}{\left(T_{p,i}-T_{p,average}\right)}^{2}}}$$

where *T_p,i_* is the predicted output, *T_a,i_* represents the experimental outputs, *T_a,average_* is the average experimental results, *T_p,average_* is the average predicted results, and n is the number of runs. The singularity concept for the model is achieved through the smallest MSE with the largest R^2^ possible [33].

### 2.12. ANOVA Statistical Analysis

All models developed were then subjected to additional analysis using analysis of variance (one-way ANOVA). Statistical significance was set at *p* < 0.05 using MINITAB by assessing the lack of fit, R-squared values (R^2^, adjusted R^2^), prediction error sum of squares (PRESS), and coefficient of variations (CV). The F-test is defined as the ratio between groups means square values within group square values and *p* values are used to investigate the significance of each coefficient. Therefore, if the *p* value is less than 0.05, the value represents a high level of significance for the associated coefficient [34]. Furthermore, analysis of variance (ANOVA) is essential in verifying the model’s performance adequacy [35].

### 2.13. Sensitivity Analysis

In developing the analysis, similar input parameters were chosen as in previous sections. To verify the effect of each parameter, the sensitivity analysis was well constructed to give a knowledge of the impact of each parameter from the ANN standpoint [36]. The sensitivity analysis was performed using the Garson equation, in which the importance of input parameters over the output parameters was determined based on the following equation:

(7) $$I_{j}=\frac{\sum_{m=1}^{N_{h}}\left(\left(\left|W_{jm}^{ih}\right|/\sum_{K=1}^{N_{i}}\left|W_{km}^{ih}\right|\right)\left|W_{mn}^{ho}\right|\right).}{\sum_{k=1}^{N_{i}}\left[\sum_{m=1}^{N_{h}}\left(\left(\left|W_{jm}^{ih}\right|/\sum_{K=1}^{N_{i}}\left|W_{km}^{ih}\right|\right)\left|W_{mn}^{ho}\right|\right).\right]}$$

where each *w* is the weight of the connection, respectively, of each *N_i_* and *N_h_*, which are the numbers of input and hidden neurons. The input parameter, *I_j_* is the relative importance (%) of the input variable j over the output variable. The superscripts ‘*i*’, ‘*h*’, and ‘*o*’ refer the input, hidden, and output layers, whereas the subscripts ‘*k*’, ‘*m*’, and ‘*n*’ refer to the input, hidden, and output neurons, respectively. A high relative importance value indicates the impact of the selected input on the output value.

## 3. Results and Discussion

### 3.1. Development of ANN with K-Fold Cross Validation

Based on the authors’ previous studies, the neural network consisted of three input nodes and three output nodes with a topology configuration of 3-8-2-3. The development of the ANN was based on the Levenberg–Marquardt learning algorithm with a hyperbolic tangent sigmoid transfer function [28]. Based on the validation neural network results, the neural network design recorded a value of 0.064 for MSE and an R^2^ value of 0.855 [37].

### 3.2. Design of Experiments and Validation Optimization of PSO Parameters

Based on the proposed 2^k^ as described in Table 2, the PSO–ANN neural network was tested in five trial runs and the average MSE readings were calculated to ensure trend consistency in data collection. ANOVA analysis was performed to determine the significance of both algorithm starting parameters, where *p* values were used to investigate the significance of each coefficient. A *p* value of less than 0.05 indicated that the associated coefficient was highly significant. According to Table 2, the PSO factorial design produced the lowest average MSE reading of 0.025 with the parameters C_1_ = 4, C_2_ = 0, and number of particles = 100.

### 3.3. Effect of PSO Parameters on Fitness Value and Optimization Process

**Figure 4:**
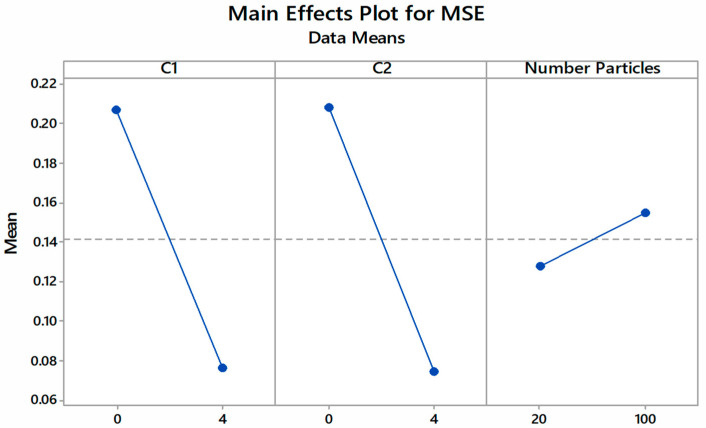
Main effects of PSO parameters against fitness value.

**Figure 5:**
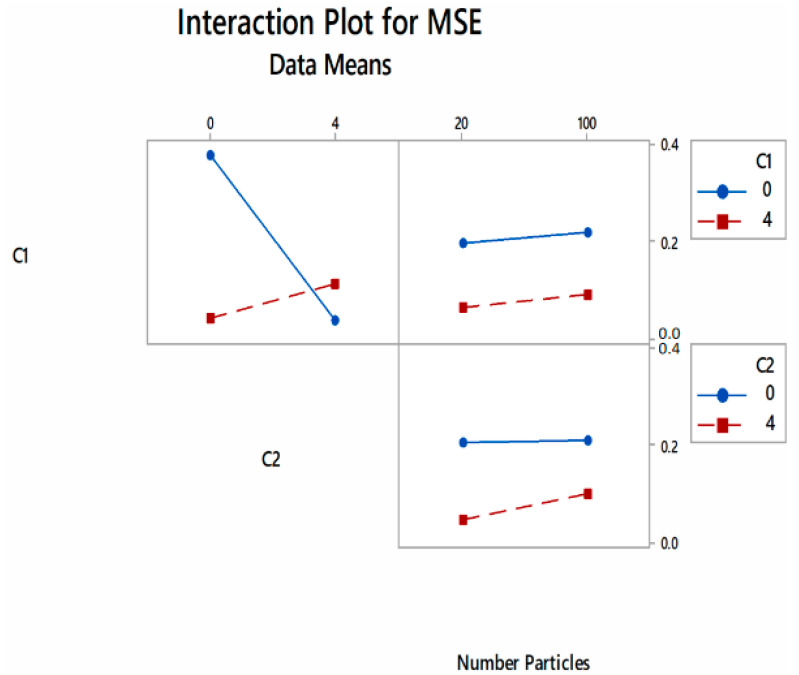
Interaction effects of PSO parameters against fitness value.

From Figure 4 and Figure 5, increasing the values of C_1_ and C_2_ resulted in a sharp drop in fitness value. When increasing the number of particles from 20 to 100, the cost function value rose, albeit at a slower rate. The use of a large number of particles in PSO optimization has resulted in comparably low MSE values, such as in the modeling study of guava drying using PSO and ANN [26]. Using a large number of particles did not improve the accuracy of the PSO based on the optimization of anaerobic wastewater treatment [38]. Furthermore, a large number of particles may increase computation time and decrease the reliability of the searched optimum value [39]. It was also shown that, at a low acceleration constant, the neural network produced a slower learning rate at lower convergence, achieving the lowest MSE values at higher iterations. Similarly, increasing the acceleration constant to a certain level led towards the ability of the network to achieve the lowest MSE value at shorter iterations [40].

The PSO parameters were then optimized based on the minimization of the fitness function (cost function), as mentioned in Equation (5), using MINITAB statistical program. Using a 2^k^ factorial design, eight sets of proposed PSO parameters with their corresponding MSE values were tested with constraints placed for each parameter to ensure that the program’s search optimization algorithm did not diverge from the search space. Table 3 shows the results of PSO parameter optimization where the optimized parameter consisted of the acceleration constant for global best (C_1_) at 4.0, the acceleration constant for personal best (C_2_) at 0, and the number of particles at 100. The parameters proposed by MINITAB suggested the lowest MSE value, which was 0.025.

### 3.4. Validity of PSO Parameters

The validation of the PSO parameters was then checked using different statistical methods. Analysis based on degrees of freedom (DF), sum of squares (SS), and mean square (MS), in which lower values of SS signified less deviation from the data and produced the best fits. The *p* value indicates the significance of effect, and the F-statistic was used for the significance test which is summarized in Table 4. Both tests were significant in the validation of the PSO parameters, in which the *p* values were less than 0.05, the F-value itself represents its own high significance among the variables. The acceleration constant for personal best (C_2_) resulted in the highest F-value (8.24), followed by acceleration constant for global best (C_1_) (1.65), and lastly the number of particles (1.37). All three F-values were supported by the significance of the *p* value, which was less than 0.05.

### 3.5. Development of PSO–ANN

**Figure 6:**
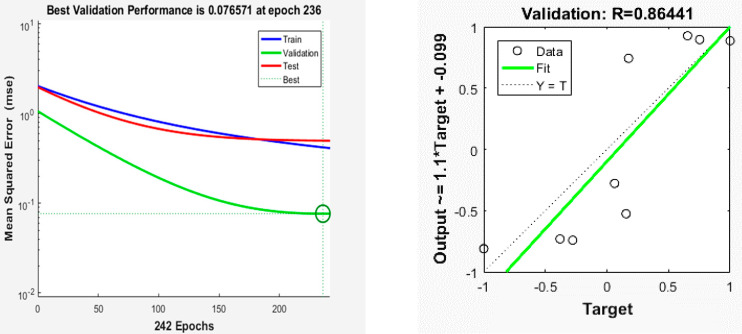
MSE value and R^2^ results of PSO-ANN.

The optimized PSO parameter was inserted into the neural network development code with an optimum configuration set with a topology of 3-2-8-3, transfer function of logsig, and a Levenberg–Marquardt algorithm, which yielded better performance with minimum MSE and highest R^2^ value. Figure 6 shows the results of MSE values and R^2^ values tabulated using MATLAB. Comparisons were made between this PSO–ANN, GA–ANN [29], and ANN [28] using their MSE and R^2^ values. The results showed that GA–ANN’s MSE was the lowest (0.033) compared with the PSO–ANN value (0.077) and the ANN value (0.082). GA–ANN’s R^2^ value was the highest (0.88) compared to PSO ≠ ANN (0.86) and ANN (0.85). This showed that PSO–ANN had a better prediction ability in comparison to the standalone ANN, whereas GA–ANN outperformed both neural models in prediction capability. GA–ANN outperformed ANN in modeling the drying process of guava pieces [26], whereas PSO–ANN was shown to outperform ANN in the recognition of citrus fruits [41].

### 3.6. Sensitivity Analysis

**Figure 7:**
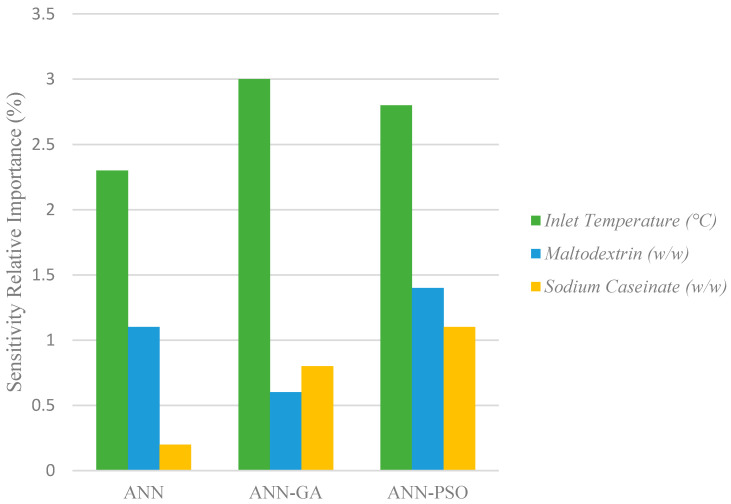
Sensitivity analysis of input variables.

Sensitivity analysis was performed on the three hybrid neural models (ANN, GA–ANN, and PSO–ANN) using three input parameters, which were the inlet temperature, concentration of maltodextrin, and concentration of sodium caseinate (Figure 7). The sensitivity analysis of each parameter provided the significance level of all three models, therefore determining the overall usefulness of the parameters on spray drying effectiveness [17].

The sensitivity analysis application from the Garson equation indicated that weight values from the input neurons were higher than the weight values for output neurons, leading towards a higher sensitivity value. Figure 7 shows that the inlet temperature was the most sensitive parameter, in all three models, to the changes in coconut milk powder quality, followed by the concentration of maltodextrin and the concentration of sodium caseinate. This was corroborated by findings that showed the inlet air temperature had a high correlation with the outlet temperature and high temperature resulted in higher moisture content extraction from the powder, leading towards a lower powder moisture content [41,42]. Greater difference in air temperature and atomized particles led to a higher evaporation rate as hot dry air constitutes at very low relative humidity [5]. The spray drying process produces heated air (inlet temperature) that directly contacts with liquid droplets, the heat required to vaporize the moisture comes from the sensible heat. Therefore, the inlet temperature will reduce and exit as outlet temperature [43]. The influence of both maltodextrin and sodium caseinate is nonetheless critical in the process of spray drying coconut milk. Studies have shown that maltodextrins provide stability to the powder formed as the glass temperature transition of the powder increased proportionally to prevent powder stickiness, whereas sodium caseinate provides stability and flowability of the formed powder [44,45].

## 4. Conclusions

A neural network built with K-Fold cross validation and the topology of the Levenberg–Marquardt learning algorithm, hyperbolic tangent sigmoid transfer function, and the 3-8-2-3 topology configuration was further integrated with the PSO algorithm for optimization of neural weights. Using a 2^k^ factorial design, three parameters of the PSO algorithm were optimized, namely acceleration constant for global best and personal best, and number of particles, and further validated using one-way ANOVA. The optimized parameters, which were 4.0, 0, and 100, respectively, were integrated into the development of the PSO–ANN. The PSO–ANN recorded MSE values of 0.077 and R^2^ of 0.86. The highest R^2^ and lowest MSE values were compared among PSO–ANN, GA–ANN, and ANN that further proved that PSO–ANN outperformed the ANN but not the GA–ANN. However, in terms of the sensitivity analysis, the PSO–ANN had the highest relative importance in the maltodextrin and sodium caseinate percentages.

## Figures and Tables

**Table 1:** Summary of PSO parameters used.

Parameter	Low	High	Significance
Acceleration constant for global best (C_1)_	0	4	Stochastic acceleration that pulls the particle towards global best position of the swarm
Acceleration constant for personal best (C_2)_	0	4	Stochastic acceleration that pulls the particle towards personal best position of the particle
Number of Particles	20	100	The number of particles in the search space

**Table 2:** PSO factorial design results based on 2^3^ level design.

PSO Parameters			Average MSE Reading	*p*-Value
Acceleration Constant for Global Best (C_1)_	Acceleration Constant for Personal Best (C_2)_	Number of Particles		
4	4	100	0.150	*p* < 0.05
0	4	100	0.045	*p* < 0.05
4	0	100	0.025	*p* < 0.05
0	4	20	0.030	*p* < 0.05
0	0	20	0.357	*p* < 0.05
4	0	20	0.055	*p* < 0.05
4	4	20	0.068	*p* < 0.05
0	0	100	0.394	*p* < 0.05

**Table 3:** Results of optimization of PSO parameters.

	Acceleration Constant for Global Best (C_1)_	Acceleration Constant for Personal Best (C_2)_	Number of Particle
**Constraints**	0–4.0	0–4.0	20–100
**Optimized PSO parameter**	4.0	0	100

**Table 4:** ANOVA results for PSO factor.

Factor		Type		Level	Values
Acceleration Constant for Global Best (C_1)_		Fixed		2	0.0, 0.4
Acceleration Constant for Personal Best (C_2)_		Fixed		2	0.0, 0.4
Number of Particles		Fixed		2	20, 100
**Analysis of Variance for Fitness Value**					
**Source**	**DF**	**SS**	**MS**	**F**	**P**
Acceleration Constant for Global Best (C_1)_	1	0.0341	0.0341	1.65	0.0151
Acceleration Constant for Personal Best (C_2)_	1	0.0513	0.05123	8.24	0.0278
Number of Particles	1	0.0033	0.0033	1.37	0.0412
